# Flexible asymmetric supercapacitors based on ultrathin two-dimensional nanosheets with outstanding electrochemical performance and aesthetic property

**DOI:** 10.1038/srep02598

**Published:** 2013-09-06

**Authors:** Shan Shi, Chengjun Xu, Cheng Yang, Yanyi Chen, Juanjuan Liu, Feiyu Kang

**Affiliations:** 1 Graduate School at Shenzhen, Tsinghua University, Shenzhen 518055, China; 2 School of Materials Science and Engineering, Tsinghua University, Beijing 100084, China; 3 School of Chemical Engineering & Technology, Tianjin University, Tianjin 300072, China

## Abstract

Flexible asymmetric supercapacitors with excellent electrochemical performance and aesthetic property are realized by using ultrathin two-dimensional (2D) MnO_2_ and graphene nanosheets as cathode and anode materials, respectively. 2D MnO_2_ nanosheets (MSs) with a thickness of ca. 2 nm are synthesized with a soft template method for the first time, which achieve a high specific capacitance of 774 F g^−1^ even after 10000 cycles. Asymmetric supercapacitors based on ultrathin MSs and graphene exhibit a very high energy density up to 97.2 Wh kg^−1^ with no more than 3% capacitance loss after 10000 cycles in aqueous electrolyte. Most interestingly, we show that the energy storage device can have an aesthetic property. For instance, a “Chinese panda” supercapacitor is capable of lighting up a red light emitting diode. This work has another, quite different aspect that a supercapacitor is no longer a cold industry product, but could have the meaning of art.

Two-dimensional (2D) nanosheets with one or several layers of atom or crystallites, such as graphene, have been investigated extensively as a new class of nanoscale materials^1^,^2^,^3^,^4^,^5^,^6^. 2D shape with an ultrathin thickness always shows relatively large surface areas and distinctive properties. It is well known that the process of the charge storage involves the insertion/extraction of protons or ions in the first few nanometers on the surface of electrode materials for pseudocapacitors and the adsorption/desorption of ions forming at the interface between electrode and electrolyte for electrochemical double-layer capacitors (EDLC)^7^,^8^,^9^. Therefore, the design of asymmetric supercapacitor based on 2D nanosheet materials with an ultrathin thickness can definitely decrease the diffusion length of ions, increase the contact area with electrolyte as well as improve active material utilization, which leads to an enhanced electrochemical performance. Manganese dioxides have attracted considerable interest utilized in asymmetric supercapacitors attributing to its low cost, abundance, and excellent electrochemical performance in aqueous electrolytes^10^,^11^,^12^,^13^. In addition, the graphene is widely investigated as the electrode material in supercapacitor due to its 2D geometry^1^,^14^. However, it is never reported the single-dispersed 2D MnO_2_ nanosheets (MSs) or the asymmetric supercapacitors with ultrathin 2D nanosheet materials as both cathode and anode materials.

With the boost of wearable and bendable consumer electronics that have an aesthetic appeal and unique functionality, it requires that the energy storage devices show not only excellent electrochemical performance, but also flexibility and shape versatility^14^,^15^,^16^,^17^,^18^,^19^,^20^. However, the state-of-the-art flexible supercapacitors generally use the flexible electrodes in the form of carbon networks as main infrastructure to reserve the flexibility^14^,^16^,^21^,^22^,^23^,^24^. Without the carbon networks pseudocapacitive materials can barely be used to fabricate the flexible electrodes, so as to assemble the flexible supercapacitors. This causes severe limitation in choosing electrode materials. Moreover, it is also a significant challenge to fabricate the flexible electrodes with a controllable shape or size by the current techniques, which is emphatically required^17^. Furthermore future consumer electronics also need the power sources with specific amazing functions, for example with transparent or aesthetic property^25^,^26^,^27^.

Herein, for the first time, flexible asymmetric supercapacitors with excellent electrochemical performance and aesthetic property were fabricated by using two kinds of ultrathin 2D nanosheet materials (MSs and graphene) through a novel strategy (Supplementary Fig. S1). The MSs with a thickness of ca. 2 nm induce to a high capacitance of 774 F g^−1^ and good rate performance. Flexible asymmetric supercapacitors using the ultrathin 2D MSs and graphene in aqueous Ca(NO_3_)_2_-SiO_2_ gel electrolyte realized excellent electrochemical performance (such as an energy density up to 97.2 Wh kg^−1^ much higher than traditional MnO_2_ based supercapacitor and no more than 3% capacitance loss even after 10000 cycles). Most interestingly, these supercapacitors can be designed to show desirably aesthetic property, for example letters, words and patterns. It provides a versatile tool to design and fabricate the supercapacitor with a certain shape through a simple process and almost all electrode materials are available. For instance an asymmetric supercapacitor with a vivid “Chinese panda” pattern has a voltage of 2.0 V capable of lighting up a red light emitting diode (LED). Our work has another, quite different aspect that a battery or supercapacitor is no longer a cold industry product, but could have the meaning of art.

## Results

We firstly use the soft template to synthesize the ultrathin MSs. A binary system consisting of surfactant (sodium bis(2-ethylhexyl)sulfosuccinate, AOT) and water can form unique structure automatically. The hydrophobic long chain of surfactant (S) tends to intertwine from each other to force hydrophilic heads toward the water phase in a binary phase. The common phase of a binary phase is the lamellar structure (referred as G phase) as shown in Supplementary Fig. S2A. In such G phase, there are alternately extended aqueous and organic lamellae. The thickness of these lamellae is about several nanometers. The inorganic salts such as KMnO_4_ can be dissolved mainly within the aqueous region, which indicates a binary liquid lamellar structure consisting of surfactant AOT and KMnO_4_ aqueous solution. Our previous work found that KMnO_4_ can be reduced by the hydrophilic head of AOT^28^. Therefore, a new self-reacting method can be proposed in this work to produce MnO_2_ ultrathin lamellae. At first, the AOT and water are mixed to form a binary lamellar structure. Then KMnO_4_ is dissolved within the aqueous region (Ws). Finally MnO_2_ will be automatically synthesized and the growth of MnO_2_ will be restricted in the aqueous region by extra AOT to form MSs.

**Figure 1:**
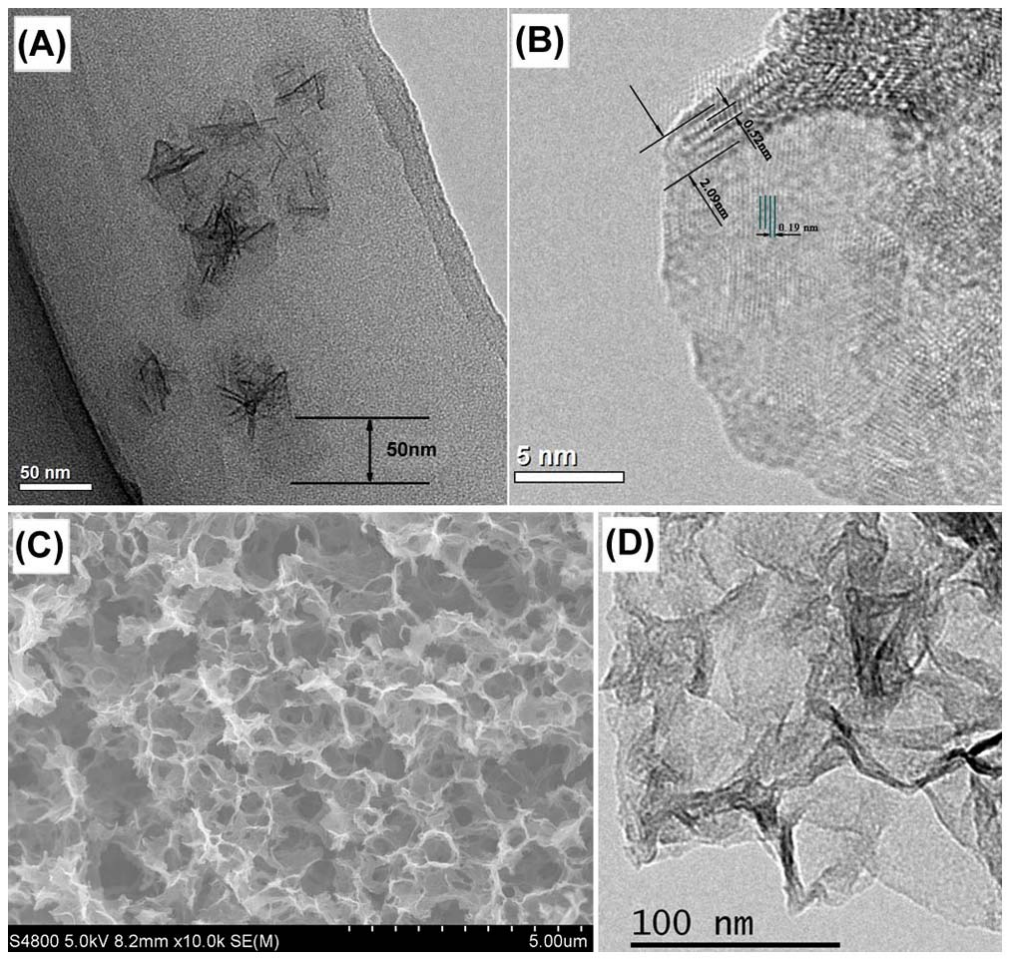
(A) TEM image of MSs, (B) HRTEM image of MSs. (C) SEM image of GA, (D) TEM image of GA.

As shown in Figure 1A, ultrathin sheet morphology is clearly presented. The average width of the MSs is ca. 50 nm. The high resolution transmission electron microscope (HRTEM) image in Figure 1B shows that the thickness of MS is ca. 2 nm, which is constructive of three or four thin flakes. Each flake has a fundamental thickness of ca. 0.6 nm corresponding to the height of [MnO_6_] octahedron known as the basic structure unit of manganese dioxide. Atomic force microscopy (AFM) study on the thickness of MS is shown in Supplementary Fig. S2B. It also indicates that MSs with ultrathin sheet morphology have an average thickness of ca. 2 nm, which is consistent with results observed in the TEM images. Well-defined sheet morphology is also clearly presented in SEM images (Fig. S2C) and a lower magnification TEM image of MnO_2_ nanosheets with a high magnification inserted are shown in Fig. S2D, which indicates a narrow size distribution of the single-dispersed MnO_2_ nanosheets. X-ray diffraction (XRD) pattern of the amorphous MSs with a few of broad peaks is shown in Supplementary Fig. S3A. MSs are further characterized by Fourier transformation infrared spectra (FTIR) and Raman spectroscopy measurement (Supplementary Figs. S3B and S3C). In the FTIR curve, the bands at frequency around 3370 cm^−1^ and 1630 cm^−1^ represent stretching and bending vibrations of water molecules or hydroxyl groups in the tunnel^29^. It is previously shown that the physisorbed water and proton facilitate the charge transfer and the diffusion of ions. Therefore, the as-synthesized amorphous MSs may lead to a better electrochemical performance compared with the crystalline form. The strong absorption observed at the lower wavenumber ranging from 800 cm^−1^ to 400 cm^−1^ corresponds to the characteristic Mn-O stretching vibrations in manganese oxides. In the Raman spectral window, the band of 580 cm^−1^ is attributed to the vibration of Mn-O in MnO_2_^29^,^30^,^31^. Porosity of MSs is also studied by N_2_ adsorption/desorption analysis (Supplementary Fig. S3D). The surface area is about 191.3 m^2^ g^−1^ calculated with the Brunauer-Emmett-Teller (BET) method, which is close to the theoretical value of 220 m^2^ g^−1^ for a MnO_2_ sheet with 2 nm in thickness and 50 nm in diameter.

The formation of graphene with continuous architecture (GA) is derived by some non-covalent bonding such as hydrogen bonding, coordination, electrostatic interaction and Π-Π stacking interactions during the hydrothermal process^32^. SEM image in Figure 1C reveals that the GA has an interconnected three-dimensional porous network with pore sizes ranging from submicrometer to several micrometers, which can substantially improve the accessible surface area and ion transport performance in electrodes. The surface area of GA calculated by BET method is as high as ca. 441.2 m^2^ g^−1^ with nitrogen absorption/desorption analysis (Supplementary Fig. S4A). TEM image in Figure 1D exhibits GA is constituted by ultrathin graphene nanosheets. The structure of graphite, graphite oxide (GO) and GA is studied by XRD (Supplementary Fig. S4B), the curve of GO shows a strong peak at 2θ = 12.2°, which corresponds to an interlayer distance of 7.2 Å due to the presence of hydroxyl, epoxy, and carboxyl groups. After hydrothermal reduction of GO for 24 h, a broad peak emerges at 2θ = 24.2° indicating the poor ordering of GA. The change of structure after reduction process is further studied by Raman Spectrum (Supplementary Fig. S4C). Both GO and GA display the existence of D and G bands located at 1352 and 1594 cm^−1^. The intensity ratio (I_D_/I_G_) of D and G bands is 0.9 in GO and increases to 1.1 in GA, which indicates some defects are introduced in GA due to the hydrothermal reduction of GO^33^.

**Figure 2:**
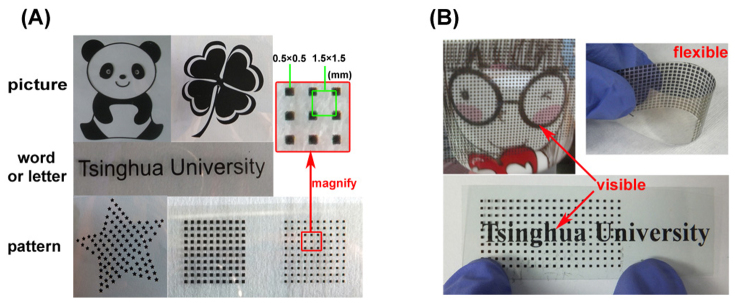
(A) Photographs of the screen-printed electrodes of various designs; (B) Photographs of electrodes demonstrating good optical transparency and mechanical flexibility. Shi Shan designed the patterns of electrodes and took the photograph.

With screen printing method, the fabricated MnO_2_ flexible electrodes can be designed as various pictures, words or letters, and patterns designed by us as shown in Figure 2A. Every fine screen-printed electrode presents a beautiful static picture. In addition, the “transparency” is controllable. For instance, in the square lattice designs the transparency increases with the size of printing pot decreases. The transparency (*T*) of electrode can be calculated by equation (1). 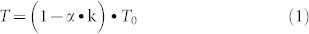
where *α* is opacity of active material film on printing stock ranging from 0 to1, *k* is rate of coverage that can be calculated by using a screen printing unit, *T_0_* is the transparency of blank unprinted sample characterized by ultraviolet and visible spectrophotometer (UV-vis). For example, toward the printed electrode with the amplified 0.5 × 0.5 (mm^2^) square lattice patterns in Figure 2A, *T_0_* is 85% (Supplementary Fig. S5), *k* is 1/9, and *α* is less than 1. Hence the transparency of electrode is no less than 76%. By controlling the degree of closeness of the printing dots the transparency of the electrode can be varied from 85% to zero (Supplementary Fig. S6). In addition, through electrodes of certain transparency, we can see the underlaid cartoon picture on a cup clearly as well as “Tsinghua University” words. Besides, the printed electrode is highly bendable and flexible as shown in Figure 2B. Note that the “transparency” discussed here means the see-through of the background visually as shown in Figure 2B.

**Figure 3:**
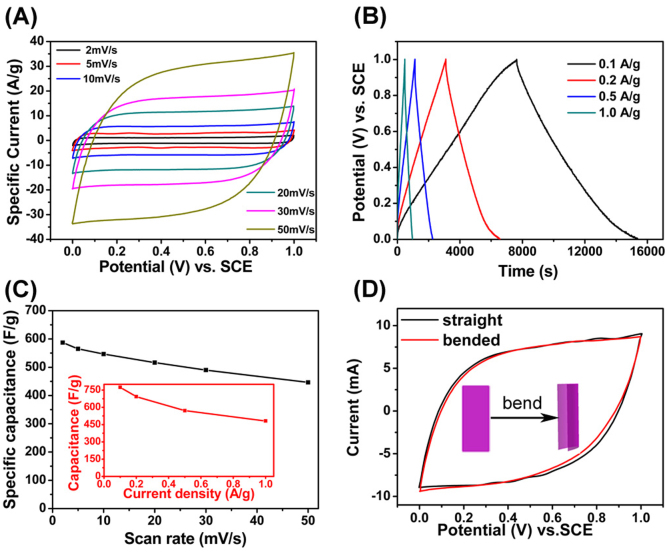
Evaluation of the performance of screen-printed MS electrode. (A) CV curves of MS electrode at different scan rates. (B) Galvanostatic charge-discharge curves of MS electrode measured with different current densities. (C) Plots of specific capacitance versus scan rate, insert is capacitance versus discharging current density. (D) CV curves of straight and bended MS electrode.

The electrochemical performance of the flexible MS and GA electrodes is investigated in a three-electrode system by using 2 molar per liter (M) Ca(NO_3_)_2_ aqueous solution as electrolyte. The Ca(NO_3_)_2_ electrolyte is used because the capacitance of MnO_2_ in aqueous electrolyte is much higher by replacing univalent cation with bivalent cation such as Ca^2+^ ion^34^,^35^. The cyclic voltammetry (CV) plots of flexible MS electrode with the scan rates ranging from 2 to 50 mV s^−1^ in Figure 3A show nearly rectangle shape, indicating that the screen-printed MS electrode has a capacitive behavior at a wide range of scan rate. The specific capacitance is 587.3 F g^−1^ at a scan rate of 2 mV s^−1^ and with about 76% of capacity retention rate at 50 mV s^−1^ (Figure 3C). According to the discharge curves presented in Figure 3B, the capacitance reaches to 774 F g^−1^ at a current density of 0.1 ampere per gram (A g^−1^) (insert of Figure 3C). It's much higher than most pure MnO_2_ nanomaterials reported in previous literature (Supplementary Table S1), although it is lower than those of some other extremely excellent MnO_2_/metal or MnO_2_/carbon materials^36^,^37^,^38^,^39^,^40^. Our ultrathin MSs also show a prominent advantage in capacitance even at fast charge rates. Because the charge storage mechanism of MnO_2_ is involved in the insertion/extraction of cations into/from the first few nanometers of electrode material, sheet morphology with an ultrathin thickness (2 nm) induces a very high capacitance because of high utilization of MSs, direct contact of sheet surface to the electrolyte and fast ion diffusion. Besides, the good contact between active materials and high conductive ITO film also facilitates the transfer of electrons. The performances of GA electrodes at a potential window of −1 ~ 0 V are presented in Supplementary Fig. S8.

In addition, the electrode is bended at 180° to further character its flexibility as shown in the insert of Figure 3D. The CV measurements have been performed on the MS electrode before (straight) and after being bended at 180°. It can be seen that two CV plots are nearly identical and the capacitance values are the same. It indicates that the MS electrode exhibits a high flexibility. The long cycle life test is performed on the flexible MS electrode at a scan rate of 100 mV s^−1^. The cycle life of the flexible MS electrode is shown in Supplementary Fig. S7. The result shows that the screen-printed MS electrode exhibits a good cycling performance with values exceeding the initial capacitance even after 10000 cycles. The ideal capacitive behavior, high specific capacitance, and good capacitance retention of screen-printed MS electrodes demonstrate that 2D ultrathin MS is one of the ideal electrode materials for supercapacitor application. The screen-printed electrode shows important advantages of high flexibility, controlled transparency and fantastic artistic design over the state-of-the-art electrodes of the flexible supercapacitors.

## Discussion

**Figure 4:**
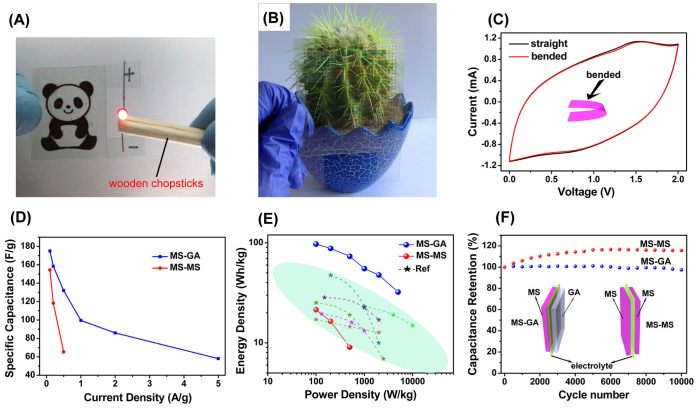
Evaluation of the performance of screen-printed supercapacitors. (A) Photograph of the “panda” asymmetric MS/GA supercapacitor lighting up a red light emitting diode. (B) Photograph of supercapacitor with a dot pattern in front of a potted plant demonstrating excellent optical transparency. (C) CV curves of straight and bended supercapacitor at 20 mV s^−1^. (D) Plots of capacitance versus current density for the asymmetric MS/GA and symmetric MS/MS supercapacitor. (E) Ragone plots of MS/GA and MS/MS supercapacitor compared with other MnO_2_ based asymmetric supercapacitors in literature (based on the total mass of active materials). (F) Cycle performance and schematic illustration of screen-printed MS/GA and MS/MS supercapacitor. The patterns of supercapacitors are designed by Shi Shan and pictures are also taken by Shi Shan.

With screen-printed MS and GA electrodes, symmetric MS/MS supercapacitors and asymmetric MS/GA supercapacitors were assembled by using a Ca(NO_3_)_2_-SiO_2_ composite gel electrolyte. The aqueous Ca(NO_3_)_2_-SiO_2_ composite gel electrolyte with high transparency and safety was synthesized by a sol-gel method (See methods in Supplementary information). The assembly of the symmetric or asymmetric supercapacitor is very simple. Two flexible electrodes are sandwiched with electrolyte. If MS and GA electrodes are used as cathode and anode, it is asymmetric MS/GA supercapacitor, while if two electrodes are both MS electrodes, it is symmetric MS/MS supercapacitor. The structures of the asymmetric and symmetric supercapacitor are shown in the insert of Figure 4F. It has to be noted that if you want to assemble the supercapacitor with the certain artwork of the flexible electrode, the pattern of the two flexible electrodes has to be symmetrical. Otherwise a misleading pattern is obtained. For example, as shown in Figure 4A MS/GA supercapacitor with a cute panda pattern designed by us is assembled by one MS electrode and one GA electrode with symmetrical panda pictures. After charging this “panda” supercapacitor up to the voltage of 2 V, it can light up a red LED (the operating voltage is 1.8 ~ 2.0 V, Supplementary Movie S1). Similarly a dot-design MS/GA supercapacitor is assembled by electrodes with symmetrical dot patterns as shown in Figure 4B. The dot-design supercapacitor is of high transparency (see-through) about 67% calculated by equation (1). Where *T_0_* is 72% characterized by UV-vis from a supercapacitor assembled by Ca(NO_3_)_2_-SiO_2_ electrolyte sandwiched with ITO-PET, where *k* is about 0.071 (Supplementary Fig. S9). Through the dot-design supercapacitor we can see the hindered ball cactus clearly as shown in Figure 4B. Most interestingly, the assembled supercapacitor also possesses preferred transparency and exquisite designs that acts not only as an energy storage device, but also as a wonderful artwork.

These screen-printed supercapacitors have been measured by the electrochemical tests. The CV measurement has performed on the MS/GA supercapacitor before and after being bended 180° at a scan rate of 20 mV s^−1^ in Figure 4C. It can be seen that CV curves of supercapacitor before and after being bended show almost the same. This result clearly shows the good flexibility of the MS/GA supercapacitor. In addition, it can be seen that CV curve exhibits a nearly rectangular shape indicating the good capacitive behavior of asymmetric MS/GA supercapacitor in the potential range from 0 to 2 V. The discharge plots (Supplementary Fig. S10) also reveal the good capacitive behavior of the MS/GA supercapacitor at different current densities. It has a specific capacitance of up to 175 F g^−1^ at a current density of 0.1 A g^−1^, and the capacitance remains 58 F g^−1^ even at a high current density of 5 A g^−1^ (Figure 4D). The Ragone plots of MS/GA supercapacitor and other MnO_2_-based asymmetric supercapacitors are shown in Figure 4E. It exhibits our 2D ultrathin nanosheets based asymmetric supercapacitor shows a much higher energy density up to 97.2 Wh kg^−1^ than those of other MnO_2_-based aqueous asymmetric supercapacitors^33^,^41^,^42^,^43^,^44^,^45^,^46^. Compared with current flexible supercapacitors based on MnO_2_/carbon (graphene, CNTs, active carbon) composites, our flexible supercapcitor based on ultrathin MSs and GA also shows relatively high energy density^16^,^18^.

We have also performed the electrochemical tests on the symmetric MS/MS supercapacitor. This MS/MS supercapacitor is also optical see-through and flexible. However, the operating voltage of the symmetric MS/MS supercapacitor is only half of the operating voltage of the asymmetric MS/GA supercapacitor. The capacitance and Ragone plots of the symmetric MS/MS supercapacitor are presented in Figures 4D and 4E, which indicates that the design of asymmetric structure highly enhances the energy density of supercapacitor.

Long cycle life tests have been performed on the symmetric MS/MS and asymmetric MS/GA supercapacitors (Figure 4F). These supercapacitors based ultrathin 2D nanosheets both show excellent cycle performance. After cycling 10000 times, the asymmetric MS/GA supercapacitor experiences less than 3% reduction in capacitance, while symmetric MS/MS supercapacitor remains more than the initial value. The increase of capacitance in the first 5000 cycles may be attributed to the active process of the electrode^47^. Similar results were frequently reported in literature^48^,^49^,^50^,^51^,^52^. In addition, it is worth to note that the MS/MS and MS/GA supercapacitors are all composed of low cost, abundant and eco-friendly ultrathin 2D nanomaterials. Furthermore, we have performed the safety measurements of the symmetric MS/MS and asymmetric MS/GA supercapacitor by cutting them into two halves at the full charge. No flash or smoke is emerged, which shows a high safety. It further confirms that screen-printed supercapacitor based ultrathin 2D nanosheets owns great promising in supercapacitor meeting present demand trends owing to the high flexibility, high-performance, shape versatility, and even transparency.

In summary, flexible asymmetric supercapacitors with excellent electrochemical performance and aesthetic property are fabricated based on ultrathin 2D nanosheets through a novel strategy. Ultrathin MSs with thickness of ca. 2 nm show a high capacitance of 774 F g^−1^ with a long cycle stability and good rate performance. Asymmetric screen-printed supercapacitors based on ultrathin 2D MSs and graphene realize good flexibility and excellent electrochemical performance, for example, a higher energy density (97.2 Wh kg^−1^) than traditional MnO_2_ based supercapacitors. Interestingly, a desirably visual and/or aesthetic property (i.e. letter, word, pattern or picture) of the supercapacitor can be realized and almost all electrode materials are available through this new strategy. This work has another and quite different aspect that the battery or supercapacitor is no longer a cold industry product, but could have the meaning of art. For example a “Chinese panda” supercapacitor is capable of lighting a LED light. We believed that it may open a new era of the flexible energy storage devices in future to meet the specific demand of the consumer electronics.

## Methods

### Preparation of ultrathin amorphous MSs

MSs were prepared by using a soft template method. Firstly, a 0.1 M sodium bis(2-ethylhexyl)sulfosuccinate aqueous solution was prepared by dissolving AOT in water and a 0.1 M KMnO_4_ aqueous solution was prepared by adding potassium permanganate (AR, 99%) in deionized water. Next 0.25 L of 0.1 M KMnO_4_ solution was added in 0.5 L AOT/isooctane solution, followed by stirring 5 h to obtain a dark brown precipitate. Then MS powders were prepared by washing the precipitates with ethanol and distilled water several times, and drying at 80°C for 12 h.

### Preparation of 3D porous graphene continuous architecture (GA)

Graphite oxide (GO) powders were initially prepared by a modified Hummers method. 160 mg GO powders was added in 80 mL deionized water followed by ultrasonication for 2 h to obtain a homogeneous graphene oxide aqueous dispersion. Then it was sealed in a 100 mL Teflon-lined autoclave and maintained at 180°C for 24 h. The autoclave was naturally cooled to room temperature and a cylindrical graphene body is self-assembled. Finally, a vacuum freeze-drying procedure was used to remove water from graphene body obtaining GA.

### Characterization

Morphologies were characterized by high-resolution transmission electron microscopy (TEM, Jeol JEM2100F), atomic force microscopy (AFM, Shimadzu, SPM9600 in tapping mode), and field emission scanning electron microscopy (SEM, Hitachi, S-4800). The structure of samples were studied by X-ray diffraction (XRD, BrukerDS RINT2000V/PC, with Cu-K_α_ radiation), Fourier transformation infrared spectroscopy (FTIR, VERTEX 70 spectrometer), and Raman spectroscopy (LabRAM HR, with an excitation wavelength of 633 nm). Nitrogen adsorption and desorption isotherms for the porosities were measured on Micromeritics ASAP 2020 surface area analyzer. Surface area was calculated by the Brunauer-Ennett-Teller (BET) method. Electrochemical tests were performed with an Im6e (Zahner) electrochemical station. All electrodes are tested by using a typical three-electrode assembly, in which a piece of platinum gauze and saturated calomel electrode were assembled as the counter and reference electrode. A mild 2 M Ca(NO_3_)_2_ aqueous solution was used as electrolyte. The capacitance values calculated from CV plots and charge-discharge curves are by following: 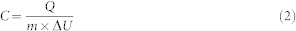


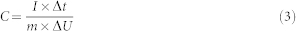
where *m* is the mass of active materials excluding binder and conductive additive, *ΔU* is the potential window, *Q* is charge, *I* is the applied current, and *Δt* is discharging time.

The energy density (E) and power density (P) of supercapacitor devices are calculated from charge-discharge curves according to following equations: 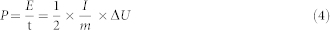

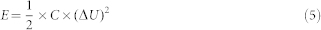
Here *I* is the applied current, *m* is the total mass of active materials, *ΔU* is the voltage of the supercapacitor, and C is the specific capacitance of the supercapacitor.

## Author Contributions

S.S. and C.X. wrote the manuscript, performed the experiments and prepared figures 1–4. C.Y. provided the screen printing method. Y.C. and J.L. synthesized the graphene. S.S., C.X. and F.K. conceived the concept and revised the manuscript. All authors reviewed the manuscript.

## Supplementary Material

Supplementary Information Supplementary Information

Supplementary Information Movie

## References

[b1] Stoller M. D., Park S., Zhu Y., An J. & Ruoff R. S. Graphene-Based Ultracapacitors. Nano Lett. 8, 3498–3502 (2008). 18788793 10.1021/nl802558y

[b2] Li D. & Kaner R. B. Graphene-based materials. Science 320, 1170–1171 (2008). 18511678 10.1126/science.1158180

[b3] Tanaka T., Fukuda K., Ebina Y., Takada K. & Sasaki T. Highly organized self-assembled monolayer and multilayer films of titania nanosheets. Adv. Mater. 16, 872–875 (2004).

[b4] Omomo Y., Sasaki T., Wang L. Z. & Watanabe M. Redoxable nanosheet crystallites of MnO_2_ derived via delamination of a layered manganese oxide. J. Am. Chem. Soc. 125, 3568–3575 (2003). 12643719 10.1021/ja021364p

[b5] Sasaki T., Watanabe M., Hashizume H., Yamada H. & Nakazawa H. Macromolecule-like aspects for a colloidal suspension of an exfoliated titanate. Pairwise association of nanosheets and dynamic reassembling process initiated from it. J. Am. Chem. Soc. 118, 8329–8335 (1996).

[b6] Xu C., Shi S., Sun Y., Chen Y. & Kang F. Ultrathin amorphous manganese dioxide nanosheets synthesized with controllable width. Chem. Commun. 49, 7331–7333 (2013). 10.1039/c3cc43055c 23851984

[b7] Diaz-Gonzalez F., Sumper A., Gomis-Bellmunt O. & Villafafila-Robles R. A review of energy storage technologies for wind power applications. Renew *&* Sust. Energ. Rev. 16, 2154–2171 (2012).

[b8] Toupin M., Brousse T. & Belanger D. Charge storage mechanism of MnO_2_ electrode used in aqueous electrochemical capacitor. Chem. Mater. 16, 3184–3190 (2004).

[b9] Frackowiak E. & Beguin F. Carbon materials for the electrochemical storage of energy in capacitors. Carbon 39, 937–950 (2001).

[b10] Xu C., Kang F., Li B. & Du H. Recent progress on manganese dioxide based supercapacitors. J. Mater. Res. 25, 1421–1432 (2010).

[b11] Mai L. *et al.* Fast Ionic Diffusion-Enabled Nanoflake Electrode by Spontaneous Electrochemical Pre-Intercalation for High-Performance Supercapacitor. Sci. Rep. 3, 1718; 10.1038/srep01718 (2013).23611904

[b12] Simon P. & Gogotsi Y. Materials for electrochemical capacitors. Nat. Mater. 7, 845–854 (2008). 18956000 10.1038/nmat2297

[b13] Xu C., Du H., Li B., Kang F. & Zeng Y. Asymmetric Activated Carbon-Manganese Dioxide Capacitors in Mild Aqueous Electrolytes Containing Alkaline-Earth Cations. J. Electrochem. Soc. 156, A435–A441 (2009).

[b14] El-Kady M. F., Strong V., Dubin S. & Kaner R. B. Laser Scribing of High-Performance and Flexible Graphene-Based Electrochemical Capacitors. Science 335, 1326–1330 (2012). 22422977 10.1126/science.1216744

[b15] Pushparaj V. L. *et al.* Flexible energy storage devices based on nanocomposite paper. Proc. Natl. Acad. Sci. 104, 13574–13577 (2007). 17699622 10.1073/pnas.0706508104 PMC1959422

[b16] Yu G. *et al.* Solution-Processed Graphene/MnO_2_ Nanostructured Textiles for High-Performance Electrochemical Capacitors. Nano Lett. 11, 2905–2911 (2011). 21667923 10.1021/nl2013828

[b17] Lee S. Y. *et al.* Progress in flexible energy storage and conversion systems,with a focus on cable-type lithium-ion batteries. Energy Environ. Sci. 6, 2414–2423 (2013).

[b18] Hu L. *et al.* Symmetrical MnO_2_-Carbon Nanotube-Textile Nanostructures for Wearable Pseudocapacitors with High Mass Loading. Acs Nano 5, 8904–8913 (2011). 21923135 10.1021/nn203085j

[b19] Hu L. *et al.* Stretchable, Porous, and Conductive Energy Textiles. Nano Lett. 10, 708–714 (2010). 20050691 10.1021/nl903949m

[b20] Hu L. & Cui Y. Energy and environmental nanotechnology in conductive paper and textiles. Energy Environ. Sci. 5, 6423–6435 (2012).

[b21] Kang Y. J., Chung H., Han C.-H. & Kim W. All-solid-state flexible supercapacitors based on papers coated with carbon nanotubes and ionic-liquid-based gel electrolytes. Nanotechnol. 23 (2012). 10.1088/0957-4484/23/6/065401 22248712

[b22] Li X. *et al.* Directly Drawing Self-Assembled, Porous, and Monolithic Graphene Fiber from Chemical Vapor Deposition Grown Graphene Film and Its Electrochemical Properties. Langmuir 27, 12164–12171 (2011). 21875131 10.1021/la202380g

[b23] Li X., Gittleson F., Carmo M., Sekol R. C. & Taylor A. D. Scalable Fabrication of Multifunctional Freestanding Carbon Nanotube/Polymer Composite Thin Films for Energy Conversion. Acs Nano 6, 1347–1356 (2012). 22236330 10.1021/nn2041544

[b24] Shi S. *et al.* Flexible supercapacitors. Particuology 11, 371–377 (2013).

[b25] Yang Y. *et al.* Transparent lithium-ion batteries. Proc. Natl. Acad. Sci. 108, 13013–13018 (2011). 21788483 10.1073/pnas.1102873108 PMC3156205

[b26] Wei D. *et al.* A Nanostructured Electrochromic Supercapacitor. Nano Lett. 12, 1857–1862 (2012). 22390702 10.1021/nl2042112

[b27] Hu L., Kim H. S., Lee J.-Y., Peumans P. & Cui Y. Scalable Coating and Properties of Transparent, Flexible, Silver Nanowire Electrodes. Acs Nano 4, 2955–2963 (2010). 20426409 10.1021/nn1005232

[b28] Xu C., Li B., Du H., Kang F. & Zeng Y. Electrochemical properties of nanosized hydrous manganese dioxide synthesized by a self-reacting microemulsion method. J. Power Sources 180, 664–670 (2008).

[b29] Sinha A. K. *et al.* Thermodynamic and Kinetics Aspects of Spherical MnO_2_ Nanoparticle Synthesis in Isoamyl Alcohol: An Ex Situ Study of Particles to One-Dimensional Shape Transformation. J. Phys. Chem. C. 114, 21173–21183 (2010).

[b30] Cho H. W. *et al.* Synthesis and supercapacitive properties of electrodeposited polyaniline composite electrode on acrylonitrile-butadiene rubber as a flexible current collector. Synth. Met. 162, 410–413 (2012).

[b31] Luo J. *et al.* Synthesis of single-crystal tetragonal alpha-MnO_2_ nanotubes. J. Phys. Chem. C. 112, 12594–12598 (2008).

[b32] Zhang L. & Shi G. Preparation of Highly Conductive Graphene Hydrogels for Fabricating Supercapacitors with High Rate Capability. J. Phys. Chem. C. 115, 17206–17212 (2011).

[b33] Gao H., Xiao F., Ching C. B. & Duan H. High-Performance Asymmetric Supercapacitor Based on Graphene Hydrogel and Nanostructured MnO_2_. Acs Appl. Mater. Interfaces 4, 2801–2810 (2012). 22545683 10.1021/am300455d

[b34] Xu C., Du H., Li B., Kang F. & Zeng Y. Capacitive Behavior and Charge Storage Mechanism of Manganese Dioxide in Aqueous Solution Containing Bivalent Cations. J. Electrochem. Soc. 156, A73–A78 (2009).

[b35] Xu C., Wei C., Li B., Kang F. & Guan Z. Charge storage mechanism of manganese dioxide for capacitor application: Effect of the mild electrolytes containing alkaline and alkaline-earth metal cations. J. Power Sources 196, 7854–7859 (2011).

[b36] Duay J., Sherrill S. A., Gui Z., Gillette E. & Lee S. B. Self-Limiting Electrodeposition of Hierarchical MnO_2_ and Mn(OH)_2_/MnO_2_ Nanofibril/Nanowires: Mechanism and Supercapacitor Properties. Acs Nano 7, 1200–1214 (2013). 23327566 10.1021/nn3056077

[b37] Yan W. *et al.* Mesoporous Manganese Oxide Nanowires for High-Capacity, High-Rate, Hybrid Electrical Energy Storage. Acs Nano 5, 8275–8287 (2011). 21942449 10.1021/nn2029583

[b38] Yan W. *et al.* Lithographically Patterned Gold/Manganese Dioxide Core/Shell Nanowires for High Capacity, High Rate, and High Cyclability Hybrid Electrical Energy Storage. Chem. Mater. 24, 2382–2390 (2012).

[b39] Lang X., Hirata A., Fujita T. & Chen M. Nanoporous metal/oxide hybrid electrodes for electrochemical supercapacitors. Nat. Nanotechnol. 6, 232–236 (2011). 21336267 10.1038/nnano.2011.13

[b40] Kim J. H., Lee K. H., Overzet L. J. & Lee G. S. Synthesis and Electrochemical Properties of Spin-Capable Carbon Nanotube Sheet/MnO_x_ Composites for High-Performance Energy Storage Devices. Nano Lett. 11, 2611–2617 (2011). 21661756 10.1021/nl200513a

[b41] Qu Q. *et al.* Electrochemical Performance of MnO_2_ Nanorods in Neutral Aqueous Electrolytes as a Cathode for Asymmetric Supercapacitors. J. Phys. Chem. C. 113, 14020–14027 (2009).

[b42] Cao J. *et al.* High voltage asymmetric supercapacitor based on MnO_2_ and graphene electrodes. J. Electroanal. Chem. 689, 201–206 (2013).

[b43] Zhang X. *et al.* Rapid hydrothermal synthesis of hierarchical nanostructures assembled from ultrathin birnessite-type MnO_2_ nanosheets for supercapacitor applications. Electrochim. Acta 89, 523–529 (2013).

[b44] Wang Y. T., Lu A. H., Zhang H. L. & Li W. C. Synthesis of Nanostructured Mesoporous Manganese Oxides with Three-Dimensional Frameworks and Their Application in Supercapacitors. J. Phys. Chem. C. 115, 5413–5421 (2011).

[b45] Jiang H., Li C., Sun T. & Ma J. A green and high energy density asymmetric supercapacitor based on ultrathin MnO_2_ nanostructures and functional mesoporous carbon nanotube electrodes. Nanoscale 4, 807–812 (2012). 22159343 10.1039/c1nr11542a

[b46] Qu Q. T. *et al.* A new cheap asymmetric aqueous supercapacitor: Activated carbon//NaMnO_2_. J. Power Sources 194, 1222–1225 (2009).

[b47] Lu X. *et al.* Facile synthesis of large-area manganese oxide nanorod arrays as a high-performance electrochemical supercapacitor. Energy Environ. Sci. 4, 2915–2921 (2011).

[b48] Feng Z. P. *et al.* MnO_2_ multilayer nanosheet clusters evolved from monolayer nanosheets and their predominant electrochemical properties. Electrochem. Commun. 11, 706–710 (2009).

[b49] Pang S. C., Anderson M. A. & Chapman T. W. Novel electrode materials for thin-film ultracapacitors: Comparison of electrochemical properties of sol-gel-derived and electrodeposited manganese dioxide. J. Electrochem. Soc. 147, 444–450 (2000).

[b50] Toupin M., Brousse T. & Belanger D. Influence of microstucture on the charge storage properties of chemically synthesized manganese dioxide. Chem. Mater. 14, 3946–3952 (2002).

[b51] Li X. & Wei B. Facile synthesis and super capacitive behavior of SWNT/MnO_2_ hybrid films. Nano Energy 1, 479–487 (2012).

[b52] Tang X., Li H., Liu Z.-H., Yang Z. & Wang Z. Preparation and capacitive property of manganese oxide nanobelt bundles with birnessite-type structure. J. Power Sources 196, 855–859 (2011).

