# Supplementary material for: Flexible asymmetric supercapacitors based on ultrathin two-dimensional nanosheets with outstanding electrochemical performance and aesthetic property
Source: Sci Rep. 2013 Sep 6;3:2598. doi: 10.1038/srep02598 (PMC3764445; doi:10.1038/srep02598)
Supplement: Supplementary Information [file srep02598-s1.pdf]

Supplementary Information (SI)

Flexible asymmetric supercapacitors based on ultrathin two-dimensional nanosheets with outstanding electrochemical performance and aesthetic property

Shan Shi[1,2], Chengjun Xu*[,1], Cheng Yang[1], Yanyi Chen [1], Juanjuan Liu[3]&Feiyu Kang*[,1,2]

[1]Graduate School at Shenzhen, Tsinghua University, Shenzhen 518055, China, [2]Department of Materials Science and Engineering, Tsinghua University, Beijing 100084, China, [3]School of Chemical Engineering & Technology, Tianjin University, Tianjin 300072, China.

Materials and Methods

1. Preparation of MnO_2 nanosheet (MS) and graphene (GA) printing ink

The MS printing ink was prepared by mixing 70wt% MS powder (synthesized with the soft template method) as active material, with 20wt% acetylene black as conductive additive, and 10wt% LA133 (purchased from Chengdu Indigo Power Sources Co., Ltd.) as binder. LA133 aqueous solution was firstly prepared by dissolving LA133 in deionized water and stirring 30min. Next acetylene black and MS were successively added in the LA133 solution under stirring. After going on stirring 8h for well dispersing, a homogeneous aqueous MS printing ink with a solid content of about 15 ±2wt% was produced.

Similarly, the GA printing ink was prepared by using 80wt% GA powder as active material with 10wt% acetylene black as conductive additive and 10wt% LA133 as binder through a process the same as preparing MS printing ink.

2. Fabrication of MS and GA electrodes

Take MS screen-printed electrodes as an example, in this work a simple manual screen printing method was utilized because of its easy operation, low cost and high quality. Firstly, a certain size of ITO-PET substrate was cut out as the printing stock and fixed on a flat and smooth table with ITO face upwards. Then a designed screen plate (the screen mesh count is 200mesh) was put on the ITO-PET with through-hole part above the ITO face. And the prepared MS printing ink was transferred onto the screen plate. Next hand a sdueegee and pressure to the screen, with a certain speed do scraping movement. With the pressure applied by the sdueegee, the ink went through the through-holes printing onto ITO film. Finally, after drying the ITO-PET printed with MS printing ink at 85 ℃ for 8h to remove solvent, the screen-printed MS electrode was obtained. By adjusting the concentration of ink, sdueegee speed, and sdueegee pressure, etc. The thickness of MS pattern can be controlled from 6 to 17 μm measured by using a micrometer. Those used in characterizing electrochemical performance usually have a thickness of about 10 μm. Typically, the loading mass of MS and GA is about 0.4 and 0.9 mg cm^{-2}, respectively.

The fabricating procedure of GA electrodes using GA printing ink with screen printing method was the same to the fabrication of MS electrodes, this electrode-made method is also suitable to most electrode materials.

3. Synthesis of aqueous $Ca(NO_3)_2$-SiO_2 composite gel electrolyte with a sol-gel method

$Ca(NO_3)_2$-SiO_2 composite gel electrolyte was prepared with a sol-gel method inspired by the ones reported in reference[1, 2]. A 2 M $Ca(NO_3)_2$ aqueous solution was firstly prepared by adding $Ca(NO_3)_2$•$4H_2O$ in deionized water. Then 90%wt of 2 M $Ca(NO_3)_2$ solution, 10wt% of SiO_2 powder (as gelating agent) and 1wt% carboxymethylcellulose (CMC, as thickening agent) were mixed in an agate mortar followed by grinding for about 30min until the mixture became clear. After several days' standing, a highly transparent $Ca(NO_3)_2$-SiO_2 composite gel electrolyte was synthesized.

4. Fabrication of flexible screen-printed supercapacitors

Before printing, it is necessary to prepare the three key elements known as printing ink, screen plate and printing stock. The ink is prepared by dispersing powders of electrode material in water or organic solvents. Occasionally, binders and conductive additives are added to enhance the adhesivity and conductivity when necessary. In this work, the aqueous printing inks of manganese dioxide and graphene (GA) powders have been prepared. The screen plate consists of blind-holes and through-holes. The ink can be printed on the printing stock through the through-holes with certain artwork form. The through-holes can be designed as various patterns, pictures or letters, such as a cute "panda", "Tsinghua University" letters and star-shaped or dot patterns as shown in Figure 2A. This character leads to its critical role, like "the magic paint brush", in creating various vivid supercapacitors such as a "panda" supercapacitor. The printing stock can be soft paper, cloth, plastic, hard glass, ceramic, etc. As an example, a flexible and transparent indium tin oxide/polyethylene terephthalate (ITO-PET) plate is used as printing stock which functions as both current collector and outer package of the supercapacitor in this work. The structure of ITO-PET is one face of a PET plate (thickness is about 0.125 mm) uniformly coated by a thin ITO film (sheet resistance is 40 Ω sq^{-1}). The ITO layer serves as the current collector, while PET plate functions as flexible outer package. Owing to the high conductivity of flexible ITO-PET substrate, it can substitute for the outer package, metal current collector and metal lead wire used in traditional supercapacitor leading to a simplified architecture of the flexible supercapacitor.

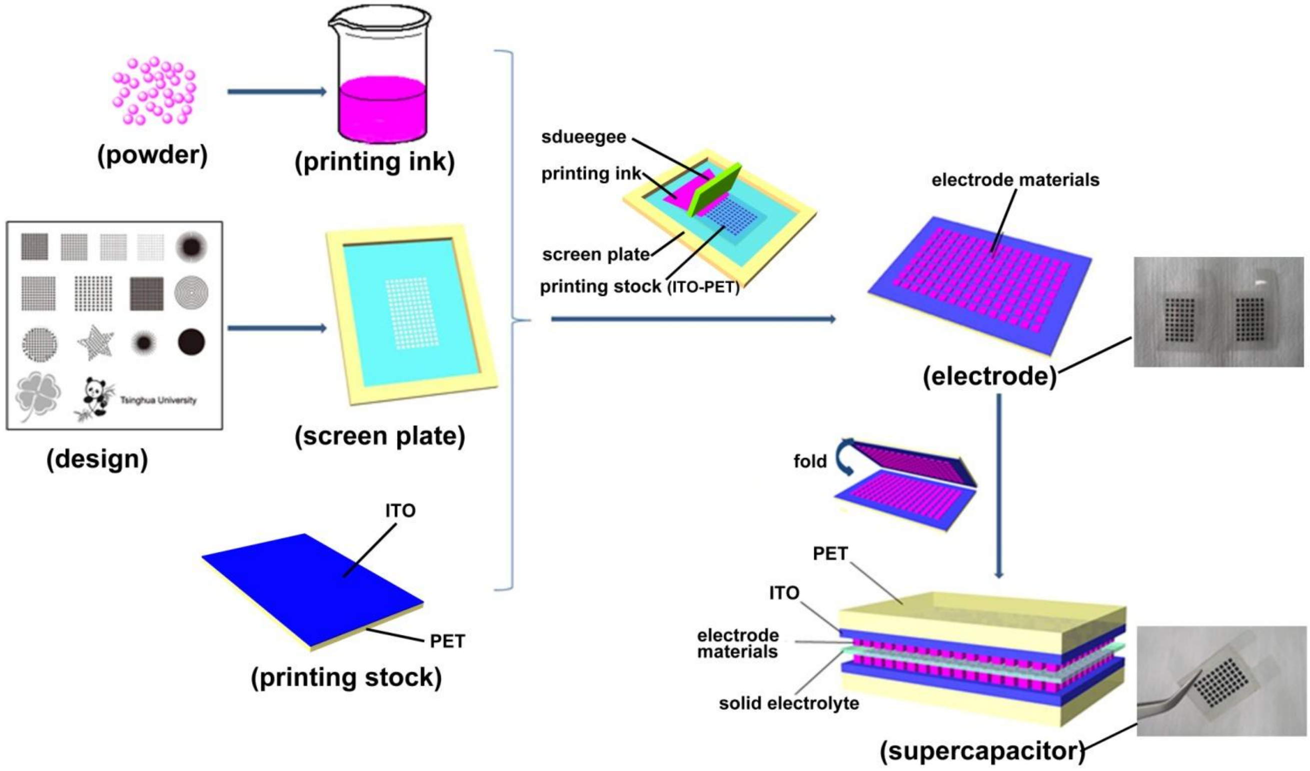

Figure S1 Schematic illustration of the fabrication of screen-printed supercapacitors. Firstly, the ink is printed on the printing stock through screen plate to prepare the flexible electrode with certain patterns. Then the two identical or different flexible electrodes with electrolyte are assembled to be symmetric or asymmetric supercapacitor.

The screen-printed supercapacitor was fabricated by two electrodes sandwiched with electrolyte, and some double-side tapes were necessary during packaging. Except for a blank ITO-PET part was setting aside to be used as the lead wire, all around the edge of the screen-printed electrode was firstly taped up with some double-side tapes. Next the window in the middle was brushed with transparent $Ca(NO_3)_2$-SiO_2 composite gel electrolyte. Then non-stick part of double-side tapes was stripped away and the other piece of electrode was folded on it. Finally, a screen-printed supercapacitor was assembled and its structure was showed in Figure S1.

It has to be pointed out that this screen printing method is suitable for inks made of almost all electrode materials in kinds of solvents, and the printing stock can be soft, hard, flat, curved as well as various shape and size. In addition, electrode materials printed on the ITO layer with especially beautiful contact are thickness-controllable and stable. Most interestingly, the versatile tool of “screen plate” provides the electrodes or supercapacitors preferred transparency and pattern. Hence, the supercapacitor presented here is not only an excellent energy storage device with flexibility but also an artwork.

Characterization

1. Characterization of MS and GA electrode materials

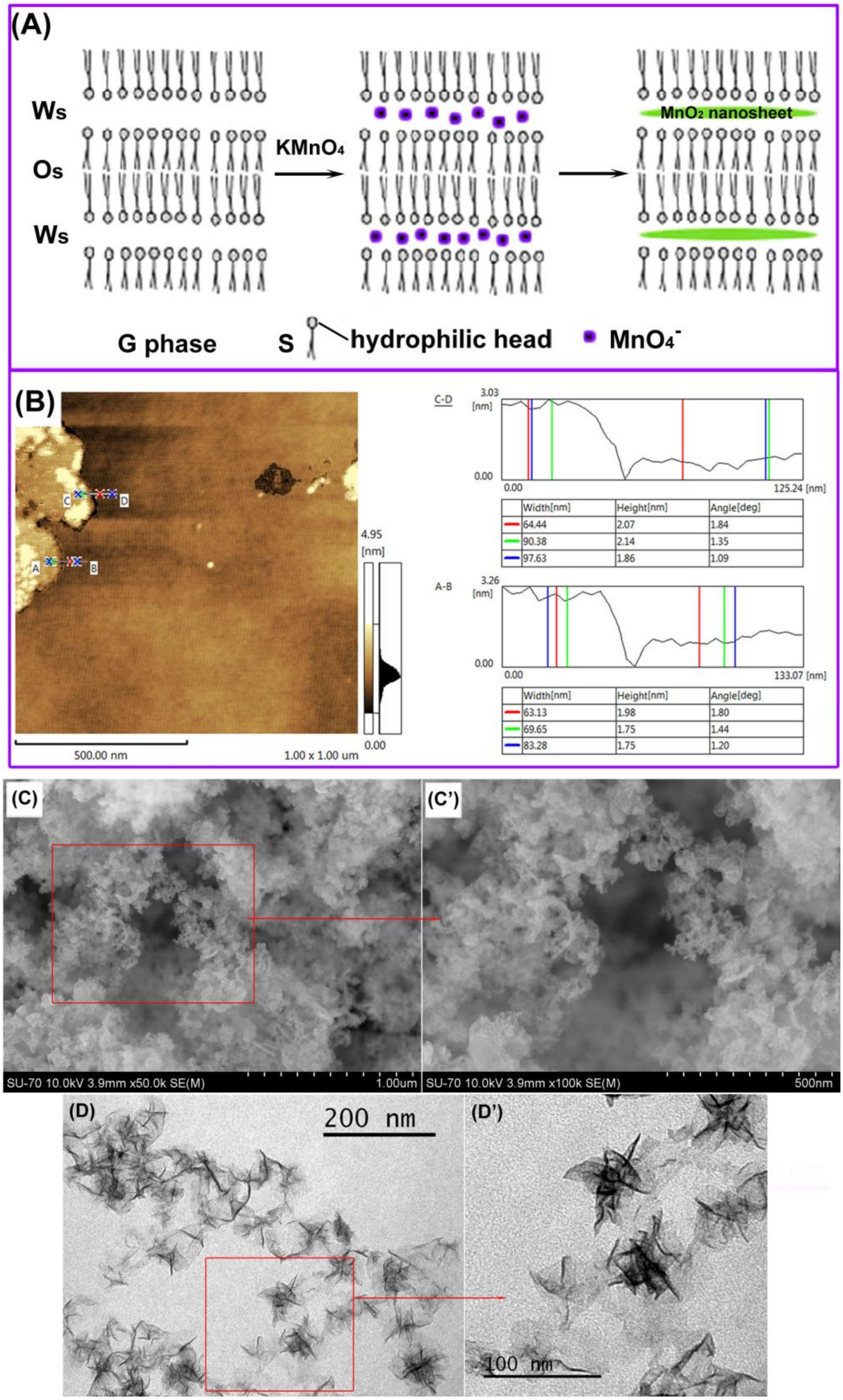

Figure S2 (A) Schematic illustration of synthesis of MSs. Os and Ws are organic and aqueous regions, respectively. S represents the surfactant AOT. The $KMnO_4$ is dissolved in the aqueous region and reduced by AOT to form MSs. (B) AFM images of MSs. (C) SEM images of MSs. (D) TEM images of MSs.

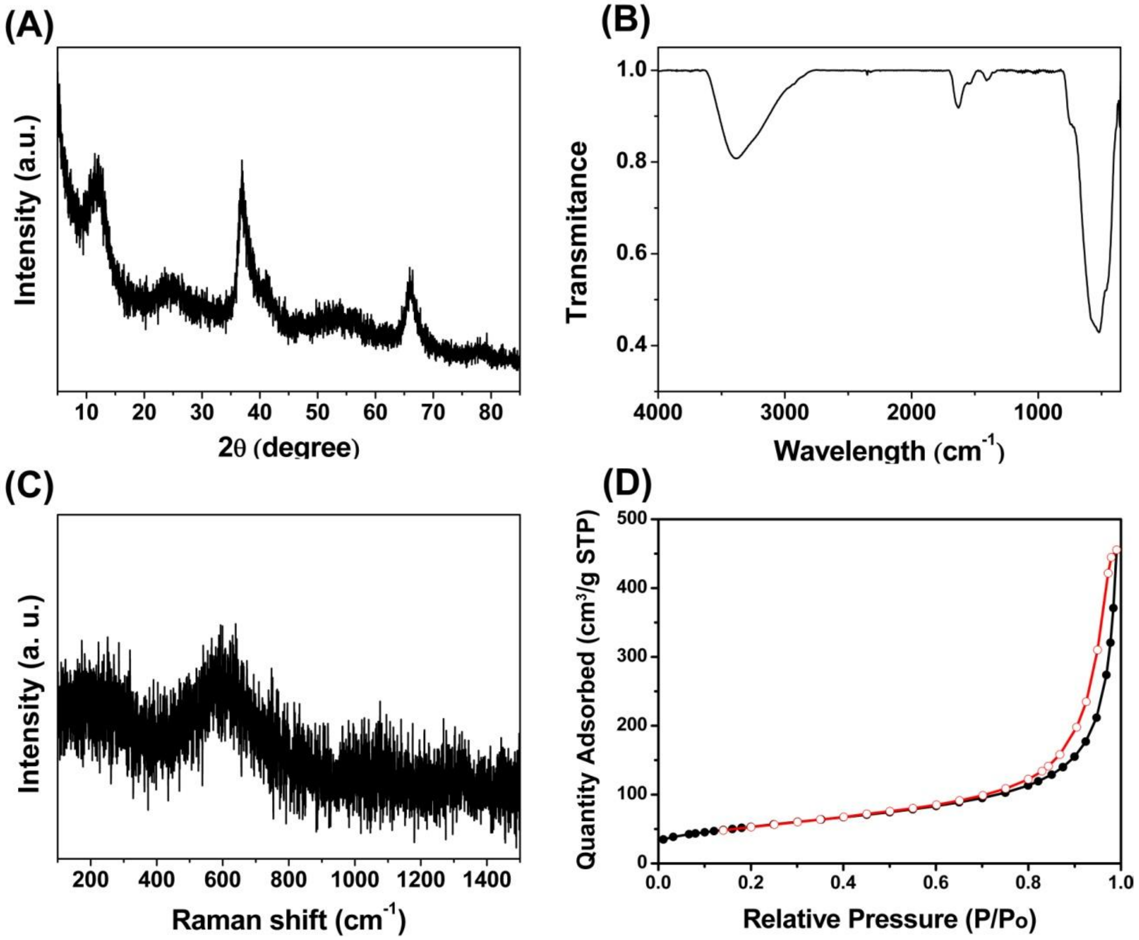

Figure S3 (A) XRD pattern of MSs, (B) FTIR spectrum of MSs, (C) Raman spectrum of MSs with 10^{-2} of the original power using an excitation wavelength of 633 nm, and (D) N_2 adsorption/desorption isotherm of MSs at 77 K.

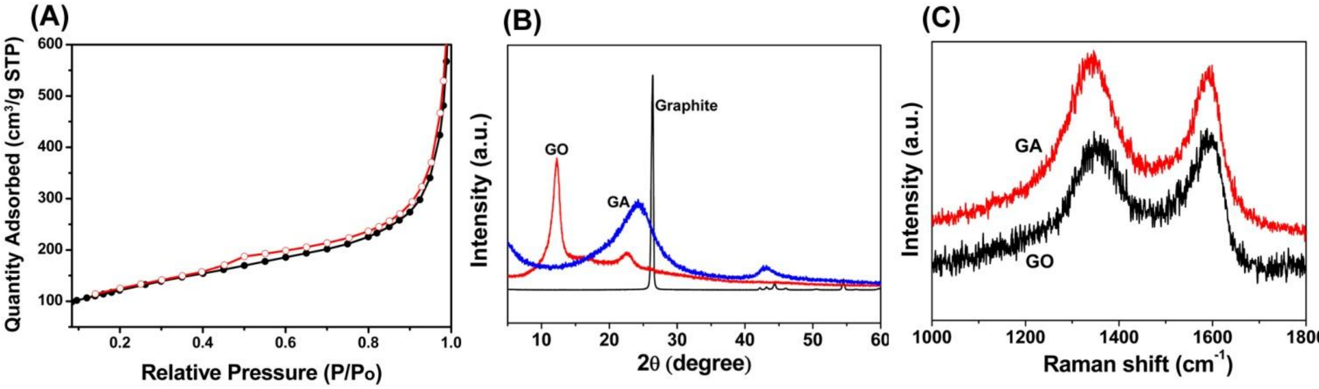

Figure S4 (A) N_2 adsorption/desorption isotherm of GA at 77 K. (B) XRD patterns of graphite, GO, and GA. (C) Raman spectra of GO and GA.

2. Characterization of screen-printed flexible electrodes

Unprinted ITO-PET substrate

The transmissivity of unprinted ITO-PET substrate is characterized by ultraviolet and visible spectrophotometer (UV-vis) (Figure S5). Based on the wavelength ranging from 450 to 800 nm, the average transparency value of unprinted ITO-PET substrate is about 85%.

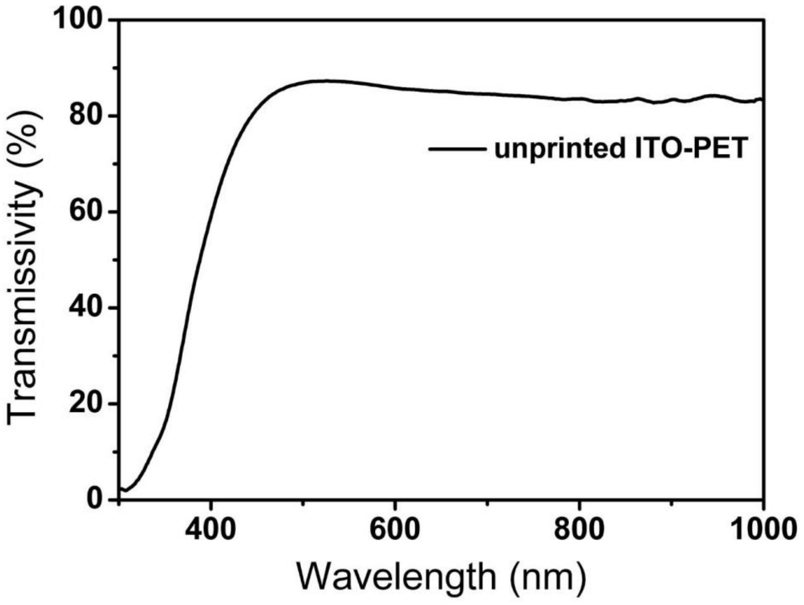

Figure S5 UV-vis spectrum of unprinted ITO-PET substrate. The average transmissivity is 85% ranging from wavelength 450 to 800 nm.

Screen-printed MS flexible electrode

Figure S6 shows the photograph of screen-printed MS electrode with different square lattice patterns. It needs to be pointed out that the four square lattice patterns (A, B, C, D) of electrode are formed by 1×1, 0.75×0.75, 0.5×0.5 and 0.3×0.3(mm^2) printed squares with an arrangement rule. And they have the same arrangement rule as the magnified one in Figure 2A. And we can readily calculate their rates of coverage: k_A =4/9, k_B =1/4, k_C =1/9, k_D =1/25. Here α is about 1.0 (the opacity of MS electrode material film printed on ITO-PET) and T_0 is 85% (transmissivity of unprinted ITO-PET presented in Figure S5). The transparency (T_C=76%) value of electrode with certain pattern in Figure S6C or Figure 2A has already been calculated by equation (1). Hence, we can calculate the transparency of electrode with the other three square lattice patterns: T_A=47%, T_B=64%, T_D=79%.

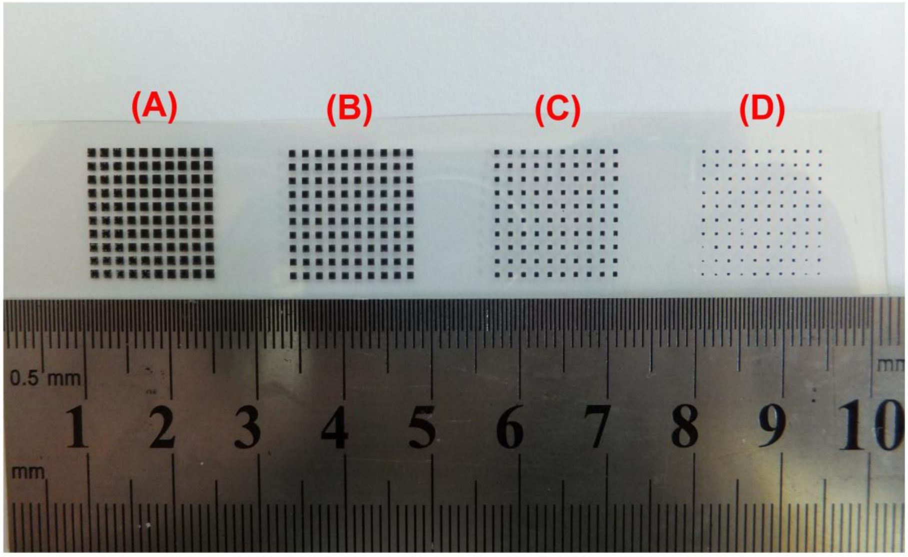

Figure S6 Photograph of screen-printed MS electrode with different square lattice patterns

Table S1 Comparison of different MnO_2-based electrodes utilizing neutral aqueous electrolyte

Morphology	Method	SC	Scan rate or	Ref

		(F g^{-1})	current density	
nanowire	hydrothermal and heat treatment	279	1 A g^{-1}	3
nanotube	hydrothermal	220	5 mV s^{-1}	4
nanorod	Sonochemistry	200	0.28 A g^{-1}	5
nanorod	electrodeposition and heat treatment	338	10 mV s^{-1}	6
layered	chemical bath deposition	447	2 mV s^{-1}	7
nanosheet	Electrodeposition	229	1 A g^{-1}	8
nanoplate	crystal growth method	404	2 mV s^{-1}	9
nanobelt	Hydrothermal	268	2 mV s^{-1}	10
nanosheet	Hydrothermal	259	0.07A g^{-1}	11
nanosheet	sonochemistry route	385	0.5 A g^{-1}	12
nanosheet cluster	electrodeposition and hydrothermal	521.5	5 mV s^{-1}	13
nanosheet	interfacial strategy	298	0.117 A g^{-1}	14
nanorod array	electrochemical deposition	660.7	10 mV s^{-1}	15
nanoflake	electrodeposition and hydrothermal	<400	0.1 A g^{-1}	16
hierarchical nanofibril/nanowire	electrochemical template method	377	50 mV s^{-1}	17
nanosheet	soft template method	774	0.1 A g^{-1}	this work
mesoporous MnO_2 on Au template	Electrodeposition	923 ± 24	5 mV s^{-1}	18
MnO_2 naosheets on Au nanowire	Electrodeposition	1020 ± 100	5 mV s^{-1}	19
nanoporous Au/MnO_2	electroless plating	1145	50 mV s^{-1}	20
MnO_2 nanopaticles on CNT	Electrodeposition	< 1250	2 A g^{-1}	21

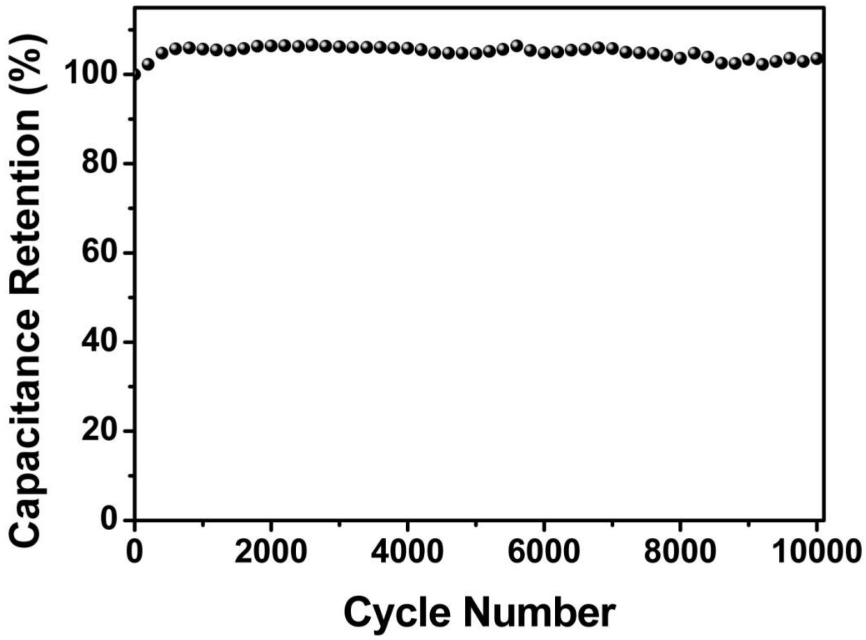

Figure S7 Cycle performance of MS electrode in 2 M $Ca(NO_3)_2$ at a scan rate of 100 mV s^{-1}.

Screen-printed GA flexible electrode

Figures S8 shows the electrochemical performance of the screen-printed GA electrode. It was measured with a

three-electrode system and 2 M $Ca(NO_3)_2$ aqueous solution was used as electrolyte. The electrodes used have a square lattice pattern with the same arrangement rule to Figure S6C (where the size of printed square point is 0.5×0.5(mm^2) and its transparency calculated by equation (1) is about 76%). They appear similar to the MS electrodes utilized in tests.

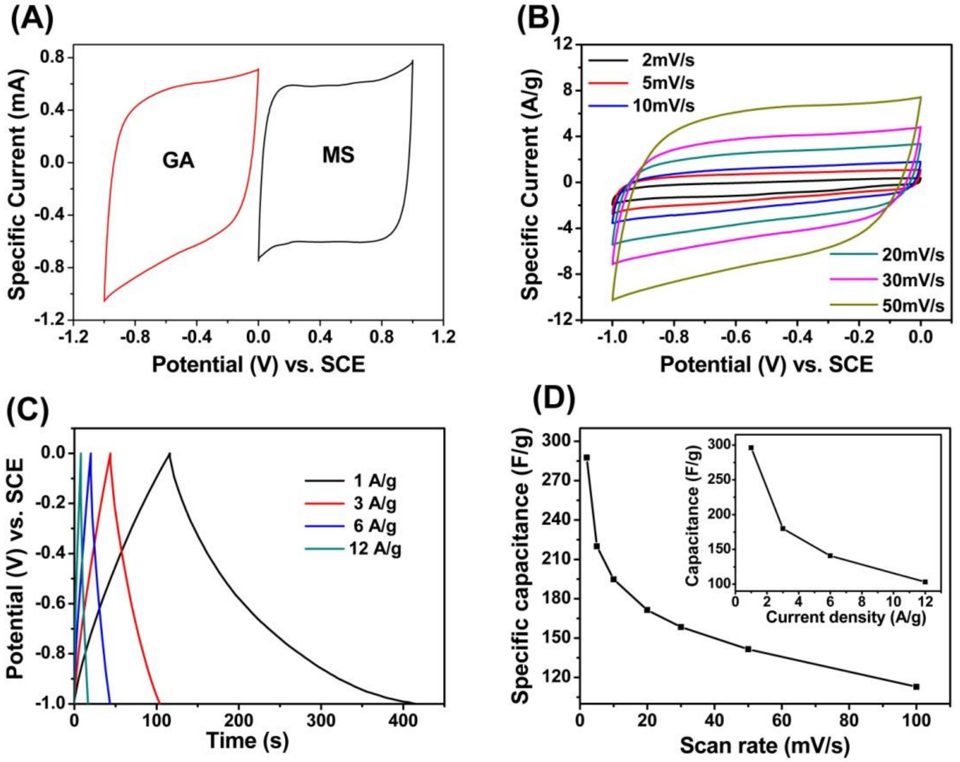

Figure S8 Evaluation of the electrochemical performance of the screen-printed GA electrode. (A) CV curves of GA and MS electrode in 2 M $Ca(NO_3)_2$ at different potential windows. (B) CV curves of the screen-printed GA electrode at different scan rates ranging from 2 mV s^{-1} to 50 mV s^{-1}, where the observed rectangular curves indicate its outstanding capacitive behavior in the potential window ranging from -1.0 V to 0 V. (C) Galvanostatic charge-discharge curves of the screen-printed GA electrode measured with current densities of 1 A g^{-1} and 12 A g^{-1}. (D) Plots of specific capacitance versus scan rate for GA, insert is versus discharging current density.

3. Characterization of screen-printed supercapacitors

Unprinted supercapacitor

Figure S9 presents the UV-vis spectrum of unprinted supercapacitor assembled with two ITO-PET substrate and $Ca(NO_3)_2$-SiO_2 composite gel electrolyte. The average transmissivity of this unprinted supercapacitor is up to 72%.

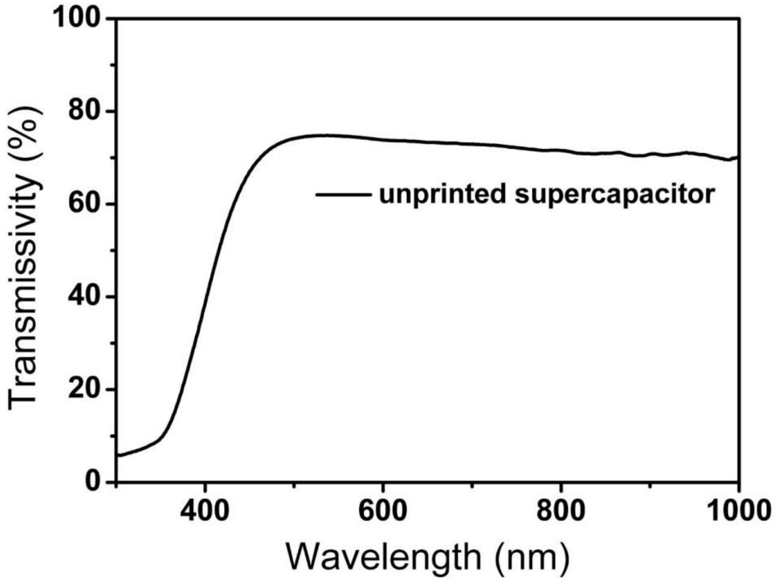

Figure S9 UV-vis spectrum of unprinted supercapacitor. The average transmissivity is 72% ranging from wavelength 450 to 800 nm.

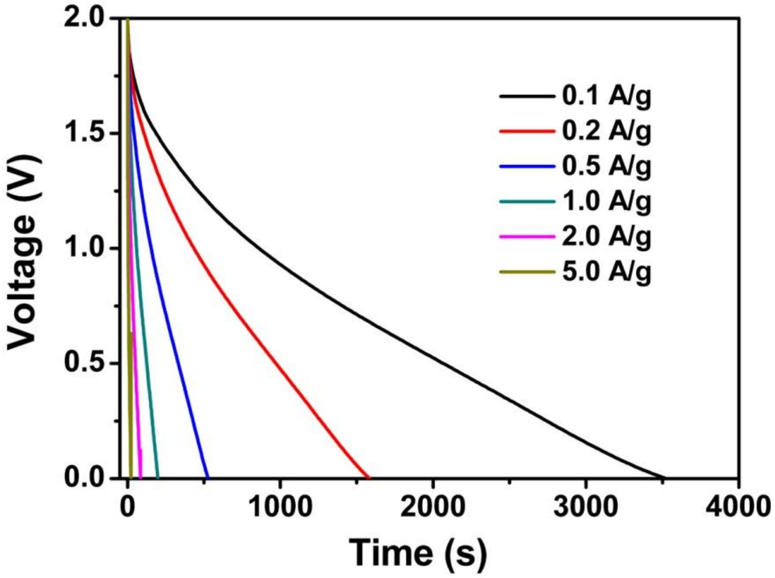

Figure S10 Galvanostatic discharge curves of the MS/GA supercapacitor measured with different current densities

Movie S1

In this movie an asymmetric MS/GA supercapacitor with "panda" design is manufactured with aqueous $Ca(NO_3)_2$-SiO_2 composite gel electrolyte. After charging the supercapacitor up to the voltage of 2V at a scan rate of 10 mV s^{-1}, it lights up a red light emitting diode (the operating voltage is 1.8V ~2.0V).

References

1. Ko, J.M. et al. Capacitive performance of the composite electrodes consisted of polyaniline and activated carbons powder in a solid-like acid gel electrolyte. *Electrochim. Acta* **50**, 873-876 (2004).
2. Moser, F. et al. Transparent electrochemical capacitor based on electrodeposited MnO_2 thin film electrodes and gel-type electrolyte. *Electrochem. Commun.* **11**, 1259-1261 (2009).
3. Jiang, H., Zhao, T., Ma, J., Yan, C. & Li, C. Ultrafine manganese dioxide nanowire network for high-performance supercapacitors. *Chem. Commun.* **47**, 1264-1266 (2011).

4. Xiao, W., Xia, H., Fuh, J.Y.H. & Lu, L. Growth of single-crystal alpha-MnO_2 nanotubes prepared by a hydrothermal route and their electrochemical properties. *J. Power Sources* **193**, 935-938 (2009).
5. Wang, H.-Q. et al. Porous nano-MnO_2: large scale synthesis via a facile quick-redox procedure and application in a supercapacitor. *New J. Chem.* **35**, 469-475 (2011).
6. Yousefi, T., Golikand, A.N., Mashhadizadeh, M.H. & Aghazadeh, M. Facile synthesis of alpha-MnO_2 one-dimensional (1D) nanostructure and energy storage ability studies. *J. Solid State Chem.* **190**, 202-207 (2012).
7. Hu, Y., Wang, J., Jiang, X., Zheng, Y. & Chen, Z. Facile chemical synthesis of nanoporous layered delta-MnO_2 thin film for high-performance flexible electrochemical capacitors. *Appl. Surf. Sci.* **271**, 193-201 (2013).
8. Wei, W., Cui, X., Mao, X., Chen, W. & Ivey, D.G. Morphology evolution in anodically electrodeposited manganese oxide nanostructures for electrochemical supercapacitor applications-Effect of supersaturation ratio. *Electrochim. Acta* **56**, 1619-1628 (2011).
9. Wan, C., Cheng, M., Zhang, Q. & Jia, N. Preparation of MnO_2 nanostructures by controlled crystal growth and its pseudocapacitive properties. *Powder Technol.* **235**, 706-711 (2013).
10. Tang, X., Li, H., Liu, Z.-H., Yang, Z. & Wang, Z. Preparation and capacitive property of manganese oxide nanobelt bundles with birnessite-type structure. *J. Power Sources* **196**, 855-859 (2011).
11. Yang, Y. & Huang, C. Effect of synthetical conditions, morphology, and crystallographic structure of MnO_2 on its electrochemical behavior. *J. Solid State Electrochem.* **14**, 1293-1301 (2010).
12. Wang, X., Liu, H., Chen, X., Evans, D.G. & Yang, W. Fabrication of manganese dioxide nanosheet-based thin-film electrode and its electrochemical capacitance performance. *Electrochim. Acta* **78**, 115-121 (2012).
13. Feng, Z.-P. et al. MnO_2 multilayer nanosheet clusters evolved from monolayer nanosheets and their predominant electrochemical properties. *Electrochem. Commun.* **11**, 706-710 (2009).
14. Yuan, C. et al. Facile interfacial synthesis of flower-like hierarchical a-MnO_2 sub-microspherical superstructures constructed by two-dimension mesoporous nanosheets and their application in electrochemical capacitors. *J. Mater. Chem.* **21**, 16035-16041 (2011).
15. Lu, X. et al. Facile synthesis of large-area manganese oxide nanorod arrays as a high-performance electrochemical supercapacitor. *Energy Environ. Sci.* **4**, 2915-2921 (2011).
16. Mai, L. et al. Fast Ionic Diffusion-Enabled Nanoflake Electrode by Spontaneous Electrochemical Pre-Intercalation for High-Performance Supercapacitor. *Sci. Rep.* **3** (2013)..
17. Duay, J., Sherrill, S.A., Gui, Z., Gillette, E. & Lee, S.B. Self-Limiting Electrodeposition of Hierarchical MnO_2 and $Mn(OH)_2/MnO_2$ Nanofibril/Nanowires: Mechanism and Supercapacitor Properties. *Acs Nano* **7**, 1200-1214 (2013).
18. Yan, W. et al. Mesoporous Manganese Oxide Nanowires for High-Capacity, High-Rate, Hybrid Electrical Energy Storage. *Acs Nano* **5**, 8275-8287 (2011).
19. Yan, W. et al. Lithographically Patterned Gold/Manganese Dioxide Core/Shell Nanowires for High Capacity, High Rate, and High Cyclability Hybrid Electrical Energy Storage. *Chem. Mater.* **24**, 2382-2390 (2012).
20. Lang, X., Hirata, A., Fujita, T. & Chen, M. Nanoporous metal/oxide hybrid electrodes for electrochemical supercapacitors. *Nat. Nanotechnol.* **6**, 232-236 (2011).
21. Kim, J.-H., Lee, K.H., Overzet, L.J. & Lee, G.S. Synthesis and Electrochemical Properties of Spin-Capable Carbon Nanotube Sheet/MnO_x Composites for High-Performance Energy Storage Devices. *Nano Lett.* **11**, 2611-2617 (2011).
